# Recognition of Supraventricular Arrhythmias in Holter ECG Recordings by ECHOView Color Map: A Case Series Study

**DOI:** 10.3390/jcdd10090360

**Published:** 2023-08-24

**Authors:** Stefan Naydenov, Irena Jekova, Vessela Krasteva

**Affiliations:** 1 Department of Internal Diseases “Prof. St. Kirkovich”, Medical University of Sofia, 1431 Sofia, Bulgaria; snaydenov@gmail.com; 2 Institute of Biophysics and Biomedical Engineering, Bulgarian Academy of Sciences, Acad. G. Bonchev Str. Bl. 105, 1113 Sofia, Bulgaria; irena@biomed.bas.bg

**Keywords:** electrocardiogram, Holter ECG monitoring, arrhythmia, extrasystole, atrial fibrillation, atrial flutter, tachycardia, Electrocardiomatrix, diagnostic image

## Abstract

Ambulatory 24–72 h Holter ECG monitoring is recommended for patients with suspected arrhythmias, which are often transitory and might remain unseen in resting standard 12-lead ECG. Holter manufacturers provide software diagnostic tools to assist clinicians in evaluating these large amounts of data. Nevertheless, the identification of short arrhythmia events and differentiation of the arrhythmia type might be a problem in limited Holter ECG leads. This observational clinical study aims to explore a novel and weakly investigated ECG modality integrated into a commercial diagnostic tool ECHOView (medilog DARWIN 2, Schiller AG, Switzerland), while used for the interpretation of long-term Holter-ECG records by a cardiologist. The ECHOView transformation maps the beat waveform amplitude to a color-coded bar. One ECHOView page integrates stacked color bars of about 1740 sequential beats aligned by R-peak in a window (R ± 750 ms). The collected 3-lead Holter ECG recordings from 86 patients had a valid duration of 21 h 20 min (19 h 30 min–22 h 45 min), median (quartile range). The ECG rhythm was reviewed with 3491 (3192–3723) standard-grid ECG pages and a substantially few number of 51 (44–59) ECHOView pages that validated the ECHOView compression ratio of 67 (59–74) times. Comments on the ECG rhythm and ECHOView characteristic patterns are provided for 14 examples representative of the most common rhythm disorders seen in our population, including supraventricular arrhythmias (supraventricular extrasystoles, paroxysmal supraventricular arrhythmia, sinus tachycardia, supraventricular tachycardia, atrial fibrillation, and flutter) and ventricular arrhythmias (ventricular extrasystoles, non-sustained ventricular tachycardia). In summary, the ECHOView color map transforms the ECG modality into a novel diagnostic image of the patient’s rhythm that is comprehensively interpreted by a cardiologist. ECHOView has the potential to facilitate the manual overview of Holter ECG recordings, to visually identify short-term arrhythmia episodes, and to refine the diagnosis, especially in high-rate arrhythmias.

## 1. Introduction

Arrhythmias are disorders in cardiac rhythm [1,2,3,4]. Generally, they are classified into two major groups: supraventricular (SVAs) and ventricular arrhythmias (VAs); both of these groups, particularly SVAs, include different subtypes of rhythm disorders [1,2]. The term ‘supraventricular arrhythmia’ literally indicates tachycardia, i.e., atrial rates > 100 beats per minute (bpm) at rest, the mechanism of which involves tissue from the His bundle or above [1,2,3]. Traditionally, SVAs have been used to describe all kinds of tachyarrhythmias apart from atrial fibrillation (AF) and ventricular tachycardias (VT), the latter originating from areas distal to the His-Purkinje system [1,4,5,6].

Epidemiological studies on the SVAs population are limited. In the general population, SVAs prevalence is 2.25 per 1000 persons and the incidence is 35/100,000 person-years [1,2,7]. Women have a risk of developing SVAs that is two times greater than that of men, and persons aged ≥65 years or have more than five times the risk of developing SVAs than younger individuals [3,8,9]. The electrophysiological mechanism of origination of most SVAs includes atrioventricular re-entry excitation made possible and maintained by accessory connections, so from the electrophysiological point of view, it is not, in essence, a true supraventricular rhythm [1,10,11,12,13,14]. SVAs are frequently regarded as narrow QRS-complex tachycardias because the QRS duration in many is ≤120 ms, which is a certain hallmark for the supraventricular origination of an arrhythmia [1,2,15]. In clinical practice, however, SVAs may present not only as narrow but also as wide QRS tachycardias, the latter requiring differentiation from VTs, which are life-threatening [1,16,17,18]. The widening of QRS in SVAs is due to functional or fixed branch blocks that may precede or occur during the SVA [1,7,19].

Initial evaluation of patients with suspected SVAs includes thorough medical history, physical examination, resting electrocardiography (ECG), laboratory (full blood counts, biochemistry profile, thyroid function), and transthoracic echocardiography [1,2,3,4,5,6]. Other investigations include Holter ECG monitoring, telemetric/transtelephonic monitoring or an implantable loop recorder, exercise tolerance testing, and coronary artery non-invasive/invasive evaluation (if myocardial ischemia is suspected) [1,2,4]. An electrophysiological study should be considered for a definitive diagnosis and when catheter ablation is anticipated [1,7,20].

Patients should be encouraged to seek medical assistance and recording of their ECG during episodes of palpitation/sensation of inappropriately accelerated heart rate [1,5,21]. If repeated, standard 12-lead ECG records do not reveal any arrhythmia, but the clinical symptoms still suggest episodes of a rhythm disorder, Holter ECG monitoring is recommended [2,8,22]. This investigation remains the gold standard for ambulatory ECG monitoring [1,3]. It is usually performed over 24–48 h (up to 72 h) and can record multiple ECG leads [1,6,8]. However, many SVAs are often sporadic and may not be frequent enough to be recorded even in ambulatory monitoring [1,12]. In such cases, more extended ECG monitoring is usually necessary to detect an arrhythmia. Recently, adhesive patch-type devices (APDs) have been used to detect SVAs [4,5,6,7,8,23]. Compared to a Holter-test, APDs are generally more compact and convenient for patients [4,5,23]. They have also the advantage of an extended monitoring period for up to several days, depending on the product [4,5,6,23]. Therefore, APDs could be a valuable alternative to the Holter ECG monitoring [6,7,23]. However, most APDs monitor single-lead ECG such that they can record ECG signals along a single vector [5,6,7,8]. As a result, there are concerns of over- or under-detection of arrhythmias compared to the standard Holter ECG [6,7,8,9].

Detection of SVA (i.e., non-sinus supraventricular rhythm) is not necessarily equal to establishment of a diagnosis [1,2,3,4,5,6,7,8]. Even if ECG, Holter ECG, or other monitoring devices detect a run of supraventricular tachycardia, they may fail to lead to a specific diagnosis [8,14,16]. In high-rate arrhythmias the short RR-intervals could hinder the recognition of the atrial excitation (P-wave, f-wave, F-wave, etc.) and the irregularity of heart rhythm, so the standard ECG methods could fail in determination of the arrhythmia type [1,8,17]. Additionally, many of the long-term monitoring devices record a single-lead ECG and their diagnostic performance could be suboptimal due to noisy tracings, frequent ectopic beats, or the coexistence of other tachyarrhythmias [5,6,17].

Holter manufacturers provide software diagnostic tools for automatic analysis of 24–72-h Holter ECG recordings to assist clinicians in the evaluation process of these large amounts of data [5,7]. Such assistance tools must suit the need for fast and accurate final diagnostic reports by cardiologists [24]. An important facility is the user interface that visualizes the ECG leads and detected heartbeats, however, finding transient and paroxysmal events requires careful examination of thousands of heartbeats in a page-by-page review of Holter recordings. Therefore, measurements and transformations that provide compact visual representations of Holter ECG data are needed. One-dimensional time-trends of automatically measured ECG features (e.g., RR-intervals, QT- and QTc-intervals, ST-segment shifts, T-wave variability, etc.) are the most simple review tools. Two-dimensional (2D) time- and frequency-domain representations are commonly used for heart rate variability (HRV) analysis by RR- and dRR-histograms, Poincaré plots and HRV spectrograms [25]. However, these HRV charts do not reflect the ECG waveform, as well as are either time-invariant or represent the influence of various factors of autonomic cardiac control. Therefore, they cannot be used to reliably detect the moment of transient arrhythmia onset/offset.

Back in 2010, an advanced 2D ECG transformation named ECHOView™ was first found as part of the DARWIN diagnostic tool of Schiller Medilog ECG Holter systems [26]. It has been shown to present thousands of heartbeats in a compressed 2D image format, where beat-by-beat amplitude variations are coded with side-by-side color bars. The resultant color map allows quick inspection of all beats in large ECG segments for incorrectly located P and T-waves. An example ECHOView transform of paroxysmal AF recording in [26] demonstrates how to recognize the onset of P-wave anomalies at a glance. This functionality is important to increase the cost-effectiveness of the AF screening service without the need for time-consuming heartbeat or arrhythmia analyses to detect all atrial events. However, we have not found published clinical feedback for the real-life usefulness of the ECHOView color map interface during expert ECG Holter record reading.

Instead, we have found the ECHOView-like principle in a later study by Li et al. (2015) [27] who presented the Electrocardiomatrix (ECM) color map as a new method for beat-by-beat visualization for inspection of cardiac signals in one lead of MIT-BIH arrhythmia database. In 2018–2019, interest in ECM increased, demonstrated in three studies for visual estimation of single-lead ECM expressed as qualitative impression for diminished HRV in patients before cardiac arrest [28]; effective manual identification of AF and/or atrial flutter (AFL) in MIT-BIH Atrial Fibrillation Database [29] and in telemetry data from ischemic stroke and transient ischemic attack patients [30]. Recently, two studies reported their satisfaction with effectiveness, intuitiveness, and memory saving while using ECM images for visual detection of events in recordings from congestive heart failure patients [31,32]. After 2020, the power of deep learning technology for image processing has been applied for the analysis of different kinds of ECM images. They are composed of single-lead stacked ECG segments with various lengths (1.5–3 s), including one or several sequential heartbeats, and analyzed with 2D convolutional layers for the purpose of AF detection [33,34,35] and biometric identification [36].

This study aims to investigate the usefulness of the ECHOView color map interface integrated into a commercial diagnostic tool for reading 24–72 h Holter ECG recordings for visual recognition of SVA episodes. The compressed ECG representation in a single page is important for fast Holter record review, therefore, we investigate the ratio of compressed information provided by ECHO View compared to conventional ECG trace in a standard grid, and whether this compressed representation is suitable to be used for unambiguous detection of paroxysmal SVA episodes. Justification by examples from clinical Holter ECG records is presented. The expected clinical benefit would be to increase the cost-effectiveness of the diagnostic service and improve patient care.

## 2. Materials and Methods

### 2.1. Holter ECG Clinical Study

We present data from an observational Holter ECG clinical study held at the Department of Internal Diseases “Prof. St. Kirkovich”, Medical University of Sofia, Bulgaria as a joint project together with the Institute of Biophysics and Biomedical Engineering at the Bulgarian Academy of Sciences (IBPhBME-BAS). The study was approved by the IBPhBME-BAS Ethics Committee (project identification code, date: No. 903ND, 10 September 2021). It was conducted in accordance with the Helsinki principles, guidelines for Good Clinical Practices, and local regulations. All participants signed an informed consent before enrollment in the study. The data anonymization policy was respected. An identification number was given to each patient. This number and all study data were proprietary to the principal investigator.

Patients meeting the following eligibility criteria were considered for enrolment in the Holter ECG clinical study:

- Inclusion criteria: (1) Age ≥ 18 years; (2) Clinical suspicion of recurrent paroxysmal/persistent arrhythmias: episode(s) of palpitations or/and acceleration of the heart rate/arterial pulse, reported by the patient as irrelevant to the level of physical activities or the emotional state; complaints consistent with pre-syncope/syncope; dyspnea or precordial oppression that cannot be explained by other conditions; (3) Possibility of the patients to have 24–72 h Holter ECG monitoring; (4) Signed written informed consent by the patients for participation in our study.
- Exclusion criteria: (1) Mentally disabled patients (irrespective of the reason) unable to understand or sign the written informed consent. (2) Patients unwilling to sign an informed consent for participation in our study or to have a Holter ECG monitoring for any reason; (3) Documented chronic arrhythmia (unless transient runs of another type of arrhythmia are suspected); (4) Clear alternative diagnosis (other than arrhythmias) for patient’s complaints.

**Figure 1:**
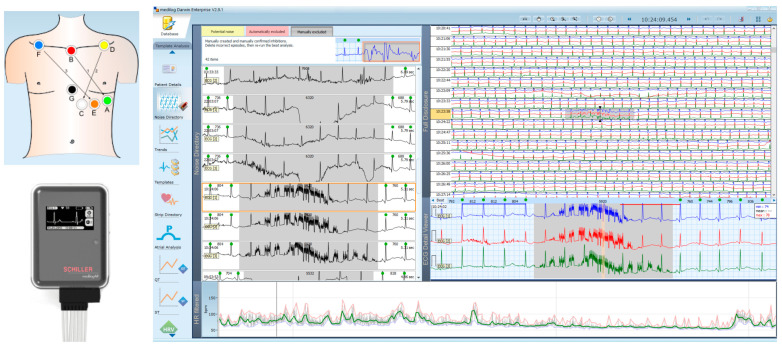
Illustration of the equipment used in Holter ECG clinical study, including: a commercial Holter ECG recorder (medilogAR 7L, Schiller AG, Switzerland) with a 7-wire patient cable [37]; Recommended placement of 7 ECG electrodes on the human chest for 3-lead configuration (ECG1 from lead vector B (red) → A (green), ECG2 from lead vector D (yellow) → C (white), ECG3 from lead vector F (blue) → E (orange)); User interface of the commercial software for Holter data revision, showing the window for noise annotation with excluded noisy episodes (in grey).

The patient’s rhythm was continuously monitored for about 24 h using a commercial Holter ECG recorder (medilogAR 7L, Schiller AG, Switzerland) with a 7-wire patient cable [37]. Three ECG leads were acquired between 7 chest electrodes placed according to the scheme in Figure 1, as recommended by the manufacturer’s instructions for use. After recording, the data were transferred from Holter device memory to PC via USB cable and stored in a database managed by commercial software for Holter ECG analysis (medilog DARWIN2 Enterprise, v2.9.1, Schiller AG, Switzerland) [37]. The automatic analysis provided by the software was run. By means of the user visual interface, all data were manually inspected and validated by an expert cardiologist (S.N) and two experienced biomedical engineers (V.K. and I.J.). In a preprocessing step, the quality of recorded data was revised using automatic labels for excluded noise and potential noise but also manually excluding noisy episodes. An example of noise annotations is illustrated in Figure 1. Common motion and noise artifacts frequently interfere with accurate rhythm assessment when ECG signals are collected from Holter monitors [38], therefore their careful exclusion is a mandatory step to ensure high-quality data for further arrhythmia analysis.

### 2.2. ECHOView Representation

**Figure 2:**
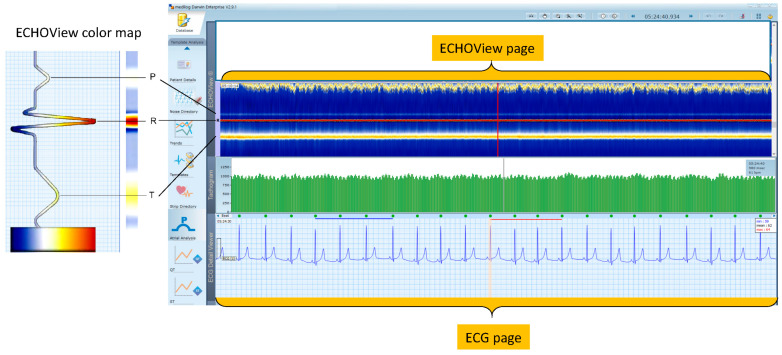
(**Left**) Principle of ECHOView color map, flattening ECG beat waveform amplitude in a color-coded bar. (**Right**) DARWIN 2 user interface reviewing a Holter-ECG recording of a patient in normal sinus rhythm. One revision page includes the following traces: (**top**) One ECHOView page built by stacked color bars of about 1740 beats in a visible pattern R ± 750 ms; (**middle**) Tachogram of 600 RR-interval time series; (**bottom**) One ECG page, including 22 s ECG signal in standard time grid (25 mm/s). Vertical lines in the three traces (ECHOView, Tachogram, ECG) point to the same time in the Holter recording. They are guides for synchronized visualization of traces, which represent different number of beats and amounts of information. All traces and markers are part of the DARWIN 2 interface except both orange highlights of the ECG and ECHOView pages.

We focus our attention on the visual interpretation of an alternative modality for ECG signal representation using ECHOView™ color maps as embedded in the user tool for “P-wave Analysis” of DARWIN2 software [26,37]. Briefly, the principle is to transform the ECG waveform amplitude into a color code so that one beat can be mapped in a single color bar as illustrated in Figure 2 (left). Due to typical amplitude differences between ECG waves, they are recognized by their different colors, e.g., red for high positive R-peaks, orange for positive T-waves, and white for low-amplitude P-waves, while negative amplitudes, if any, are scaled from blue-white to blue-black. DARWIN2 interface integrates stacked color bars of many sequential beats, particularly including about 1740 beats in one page of the ECHOView image, as shown in Figure 2 (right, top). All beats are aligned by their R-peaks, which can be recognized as a red horizontal line in the middle part of the ECHOView color map. The height of one ECHOView page is defined by the visible ECG pattern around the R-peak, which can be resized by the user to ±250 ms, ±500 ms, or ±750 ms. Our further observations are carried out in a pattern, R ± 750 ms (1500 ms in total), including the P-wave, and the T-wave but also parts of previous and next beats (depending on the heart rate). One ECHOView page displays the beat patterns in one ECG lead. The user interface gives the option to switch between the ECHOView pages of the three recorded ECG leads. The color scale is fixed by software settings and is uniformly applied to the entire record.

The careful examination of the ECG rhythm by an expert requires a manual page-by-page review of the long Holter ECG recording. Therefore, the amount of well-interpretable information in one revision page is inversely proportional to the time spent on the revision, i.e., more information in fewer revision pages results in shorter reading time. Thus, our task is to compare one ECG page vs. one ECHOView page in two aspects:

1. Interpretation capability focused on recognition of arrhythmias.
2. Compression capability expressed by compression ratio (CR) according to the number of ECG pages (nP_ECG_) and the number of ECHOView pages (nP_ECHOView_) required to review one Holter-ECG recording:

CR = nP_ECG_/nP_ECHOView_ (1)

One ECG page is viewed in a standard time grid (25 mm/s), including 22 s of ECG signal (Figure 2). Thus, nP_ECG_ is strictly defined by the duration of the ECG recording. On the other hand, one ECHOView page is composed of a fixed number of beats (Figure 2), therefore nP_ECHOView_ is defined by the number of beats in the ECG recording and thus depending on the heart rate.

### 2.3. Clinical and Diagnostic Assessment

**Figure 3:**
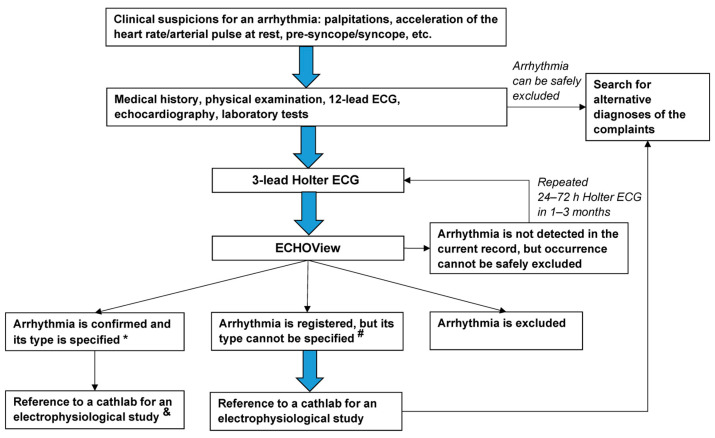
Flowchart of the diagnostic approach applied to the patients in this study. The thick blue arrows indicate the obligatory diagnostic steps. Notes: * Diagnosis is based on the current ECS guidelines for the diagnosis of supraventricular and ventricular arrhythmias [1]. ^#^ High-rate arrhythmias, artifacts, previous bundle branch block, poor contact of electrodes, loss of signal, or other factors that obscure ECG components, are essential for the diagnosis and specification of the type of arrhythmia. ^&^ If arrhythmia episodes are causing hemodynamic instability or having a high symptomatic burden and are resistant/contraindicated for pharmacological treatment/patients unwilling to take antiarrhythmics.

The diagnostic approach applied to the patients in this study is summarized in Figure 3. All individuals who attended the clinic where the study was conducted and had symptoms, consistent with an arrhythmia, underwent a thorough clinical and instrumental investigation, including medical history taking (current and previous symptoms, past medical history, ongoing and previous pharmacological treatment, risk factors, family history, etc.), followed by a physical examination, focused on the cardiovascular system, standard 12-lead ECG at rest, echocardiography and routine laboratory parameters (complete blood count, thyroid hormones, electrolytes, inflammation markers, etc.). After these initial investigations all patients in sinus rhythm, in whom an arrhythmia could not be excluded, i.e., the symptoms, clinical and instrumental pathological findings were not due to a clear alternative diagnosis, had a 24-h 3-lead Holter ECG monitoring with consecutive processing of the records in ECHOView. The initial 12-lead ECG, Holter ECG, and ECHOView records were visually inspected by a cardiologist for the presence of arrhythmias and in case of uncertainty, a second opinion was sought by another cardiologist. If no arrhythmia has been registered in the initial ECG, Holter ECG, and ECHOView records, but strong clinical suspicions for a rhythm disorder remain (persisting typical symptoms, risk factors, medical history of recorded arrhythmias, but missing documents, etc.) a second 24–72 h 3-lead Holter ECG monitoring was conducted. Additional investigations, including an invasive electrophysiological study, were performed only on those participants in whom diagnostic difficulties/uncertainties remained even after the second Holter monitoring and ECHOView, and/or if participants fulfilled the criteria for such investigations.

While manually reading Holter-ECG recordings, we applied the following diagnostic criteria to recognize normal rhythm and arrhythmia events under the scope of this study:

1. Normal sinus rhythm (NSR): rhythm in which a typical P-wave with amplitude ≥ 0.5 and <3 mm, and duration <120 ms can be found in at least 1 Holter ECG lead. The P-wave precedes the QRS at a fixed interval of 120–200 ms in any cardiac cycle. The heart rate (HR) is ≥60 bpm and ≤100 bpm.
2. Supraventricular/ventricular extrasystole (SVES/VES): a single narrow/broad QRS complex beat (early, interpolated, or late) with no identifiable or with an atypical P-wave before or after the QRS (P-wave morphology differs from the morphology of the sinus P-wave of the corresponding participant) [39].
3. Sinus tachycardia (SINT): sinus rhythm (all P-wave criteria from point 1 fulfilled) with HR > 100 bpm.
4. Supraventricular tachycardia (SVT): A narrow QRS complex tachycardia (≥3 consecutive QRS complexes with QRS duration < 120 ms and HR > 100 bpm) with no discernible sinus P-waves in any of the Holter ECG leads. If any atrial excitation is recorded, it fails the criteria for a sinus rhythm P-wave described above, i.e., P-waves may be seen, but their morphology is different from that of the sinus beat. The SVTs were further sub-classified using the criteria, proposed by the 2019 ESC Guidelines for the management of patients with supraventricular tachycardia [1].
5. Atrial fibrillation (AF): a supraventricular, usually narrow QRS complex arrhythmia (≥3 consecutive QRS complexes) with irregularly irregular rhythm (varying from beat-to-beat RR intervals with no identifiable algorithm according to which RR intervals change). No typical sinus P-waves are seen in any Holter ECG lead, just fibrillations (“f” waves) of the isoelectric line. If there is a fixed or functional bundle branch block, QRS may be ≥120 ms, but the other characteristics of the rhythm are as described above.
6. Atrial flutter (AFL): a supraventricular, usually narrow QRS complex arrhythmia (≥3 consecutive QRS complexes) with absent typical sinus P-waves and the presence of “saw-like” deflections of the isoelectric line with fixed (regular rhythm) or varying (irregular rhythm) atrioventricular conduction. If there is a fixed or functional bundle branch block, QRS may be ≥120 ms, but the other characteristics of the rhythm are as described above.
7. Ventricular tachycardia (VT): a broad QRS complex tachycardia presented in ECG with ≥3 consecutive QRS complexes with duration ≥120 ms (usually ≥140 ms) at heart rate >100 bpm. If sinus/non-sinus P-waves are visible they are not related to the QRS complexes, i.e., there is atrioventricular dissociation (randomly varying PR intervals). If the PR interval is fixed, a SVA with a bundle branch block has to be excluded. VTs were further sub-classified using the criteria, proposed by the 2022 ESC Guidelines for the management of patients with ventricular arrhythmias and the prevention of sudden cardiac death [39]. If a VT continued ≤30 s it was regarded as a ‘non-sustained VT’ (NSVT) [39].

## 3. Results

### 3.1. Overview of Patients’ Data and Holter ECG Reading

Patients were enrolled from October 2021 to December 2022. In total, 86 consecutive patients have been included, of them 41 (47.7%) males and 45 (52.3%) females, *p*-value = 0.601. The age of the study population was 56 ± 16 (23–89) years, reported as mean ± standard deviation (min–max range).

The Holter ECG recordings had a total valid duration of 21 h 20 min (19 h 30 min–22 h 45 min) counted after the removal of artifacts, which represents about 3.3% (0.8–13.7%) of the total record length, where values are reported as median (quartile range). The total number of beats reviewed per Holter ECG record was 88,862 (75,569–101,934), presenting minimal HR = 48 (43–53) bpm, maximal HR = 128 (110–149) bpm and mean HR = 71 (64–81) bpm. The number of reviewed pages was:

- 3-lead Holter ECG: nP_ECG_ = 3491 (3192–3723) pages per record.
- ECHOView: nP_ECHOView_ = 51 (44–59) pages per record.

This result makes it possible to evaluate the strong ability of ECHOView to compress information, giving CR = 67 (59–74) times less number of required revision pages than ECG. Considering the above reported median statistics for the HR mean (min–max) of 71 (48–128) bpm, we can calculate that one ECHOView page with 1740 beats includes an ECG duration of about 24.5 min (13.6–36.3 min). 

The collected Holter ECG recordings provided the opportunity to verify the ECHOView interpretation capability with the following rate of arrhythmia findings in our patient population (one patient recording could contain more than one rhythm disorder):

- NSR in 78 patients (91%);
- SVES in 72 patients (84%), in whom the appearance of SVES is not frequent, was found in a mean proportion of 0.36% (0–9.83%, min–max) from all beats. None of the patients had a clinically important SVES prevalence (>10%).
- VES in 67 patients (78%), in whom the appearance of VES is not frequent, was found in a mean proportion of 0.72% (0–11.95%, min–max) from all beats. Only one patient had a clinically significant prevalence of VES (11.95%), defined for our study as premature ventricular depolarizations ≥10% of all ventricular depolarizations.
- AF in 5 patients (5.8%), in whom the mean AF proportion was 66% (0–100%, min–max) from the total Holter ECG recording duration, detected in a mean number of 2.8 (1–9, min–max) episodes per recording;
- AFL in 5 patients (5.8%), in whom the mean AF proportion was 20% (0–76%, min–max) from the total Holter ECG recording duration, detected in a mean number of 196 (2–287, min–max) episodes per recording;
- ST and SVT in 41 patients (48%) detected in a mean number of 31 (1–669, min–max) episodes per recording;
- NSVT in 3 patients (3.5%) with very short rushes from 3 to 11 beats.

### 3.2. NSR, SVES, VES Cases

**Figure 4:**
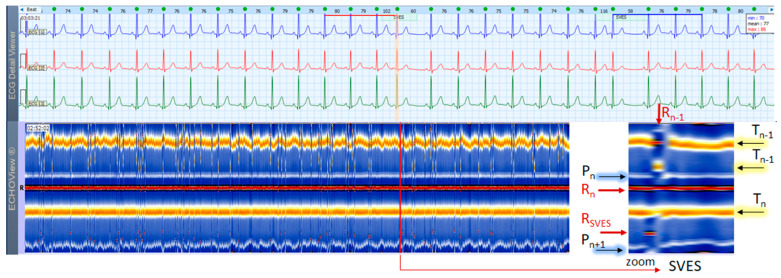
A 3-lead Holter ECG strip (22 s, 25 mm/s) and ECHOView image (ECG lead 1, height = 1500 ms, width resolution = 80 beats/25 mm) of a patient with NSR and frequent early unifocal SVES. The two pages (ECG—top and ECHOView—bottom) are aligned with vertical lines. Zooming of the ECHOView area of interest around SVES shows that the early non-sinus ventricular depolarization (R_SVES,_) is visible as a red dot in the blue horizontal area between T-waves (T_n_) and P-waves (P_n+1_) of the sinus rhythm beats; the depolarization R-peak of the sinus beat preceding SVES (R_n−1_) is visible as a red dot within the orange band of the sinus rhythm T-waves (T_n−1_); the repolarization T-peak of the sinus beat preceding SVES (T_n−1_) is visible as an orange dot within the blue horizontal area between T_n−1_ and P_n_ of the sinus rhythm beats. SVES in the not zoomed ECHOView page are recognized as occasional narrow vertical lines with different colors than the background color map presented by the NSR. P_n_, P_n+1_—P-wave of the normal atrial depolarization (P_n_: current, P_n+1_: next); R_n_—R-peak of ventricular depolarization; R_n−1_—R-peak of previous normal ventricular depolarization; R_SVES_—R peak of SVES; T_n_, T_n−1_—T-wave of the normal ventricular repolarization (T_n_: current, T_n−1_: previous); NSR—normal sinus rhythm; SVES—supraventricular extrasystoles.

**Figure 5:**
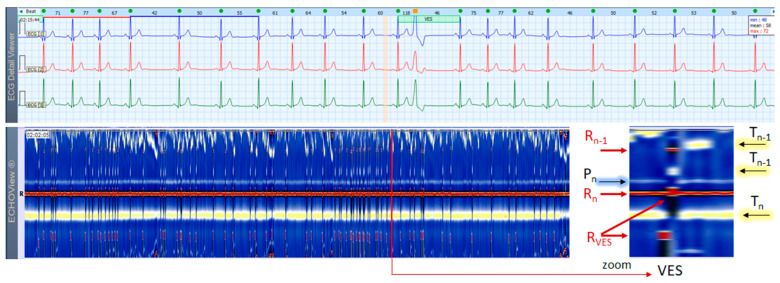
A 3-lead Holter ECG strip (22 s, 25 mm/s) and ECHOView image (ECG lead 1, height = 1500 ms, width resolution = 80 beats/25 mm) of a patient with NSR and frequent VES. The two pages (ECG—top and ECHOView—bottom) are aligned with vertical lines. Zoomed ECHOView around VES shows the early non-sinus ventricular depolarization (R_VES_) as a red band, which is thicker than (R_n_) of normal sinus depolarization due to VES wide complex (consecutive not simultaneous excitation of both ventricles). The white-blue P-wave band (P_n_) of NSR is interrupted during VES due to the lack of normal atrial excitation. The dark spot following R_VES_, spreading also on the yellow-white T-wave band (T_n_) is due to ST-segment depression and a T-wave electric axis that is opposite to the electric axis of R-wave in VES. VES in the not zoomed ECHOView page are recognized as sporadic narrow vertical lines with a different color than the background color map presented by NSR. P_n_—P-wave of the normal atrial depolarization; R_n_—R-peak of ventricular depolarization; R_n−1_—R-peak of previous normal ventricular depolarization; R_VES_—R peak of VES; T_n_, T_n−1_—T-wave of normal ventricular repolarization (T_n_: current, T_n−1_: previous); NSR—normal sinus rhythm; VES—ventricular extrasystoles.

This section interprets together 3-lead Holter ECG strips and the corresponding ECHOView images (ECG lead 1) of two patients in sinus rhythm with frequent supraventricular (Figure 4) and ventricular extrasystoles (Figure 5). Detailed interpretation of the zoomed ECHOView color map in the area of interest around one extrasystole is presented in the caption of the figures. In general, SVES and VES can be easily recognized as occasional narrow vertical lines of a different color from the background color map presented by NSR. In the zoomed ECHOView images, we can identify important SVES and VES differences vs. sinus rhythm beats, such as the early non-sinus ventricular depolarization visible as a red dot in the blue horizontal area between T-waves and P-waves (Figure 4 and Figure 5); typically for VES morphological alterations—the wider R-waves, the black spots with ST-segment depression and the T-wave electric axis inversion (Figure 5). Despite ECHOView being useful for the localization of vertical lines of extrasystoles at a glance, the ECG trace is mandatory to verify their type.

It is worth noting that all supraventricular and most ventricular extrasystoles (except R-on-T type which can trigger a VT) are not life-threatening, and if they are not extremely frequent (bi- or trigeminy), they do not worsen the patient’s hemodynamics and clinical condition. Extrasystoles do not even require treatment if they do not impair the quality of life significantly or/and if <10% of all heartbeats. For this reason, the diagnostic advantage of standard ECG/Holter ECG vs. ECHOView is not of clinical merit. What ECHOView could improve in patients with extrasystoles is the reduction of the Holter reading time, particularly saving time for a visual finding of SVES and VES events, especially valuable for records with duration > 24 h.

### 3.3. Sinus Tachycardia Cases

**Figure 6:**
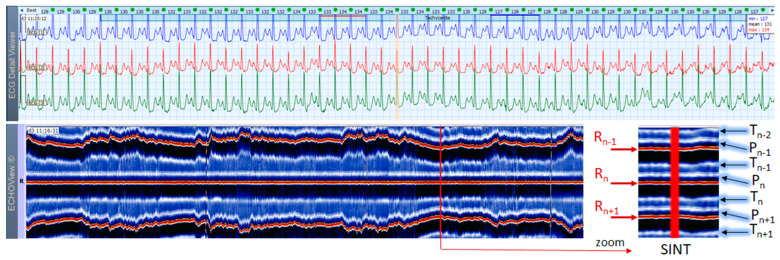
A 3-lead Holter ECG strip (22 s, 25 mm/s) and ECHOView image (ECG lead 1, height = 1500 ms, width resolution = 80 beats/25 mm) of a 46-year old female patient with SINT. The two pages (ECG—top and ECHOView—bottom) are aligned with vertical lines in the highest rate SINT (max HR = 134 bpm read from instant HR values above each QRS complex in the ECG page). In ECG, the high rate makes the P-wave to superimpose on the previous T-wave with no isoelectric line between them, so the ‘notch’ between T- an P-waves may be mistaken for a retrograde atrial excitation, found in AVRT with concealed accessory pathways and sometimes in AFL. Similar P-wave morphology may also be found in focal atrial tachycardia. In ECHOView (original and zoomed), the P-wave is a visible blue-white horizontal band (P_n_), preceding the red R-wave line (R_n_), which confirms the sinus rhythm. ECHOView color map: Blue-white traces (P-wave of atrial depolarization, P_n_: current, P_n+1_: next, P_n−1_: previous); Red traces (R-peak of ventricular depolarization, R_n_: current, R_n+1_: next, R_n−1_: previous); Blue-white traces (T-wave of ventricular repolarization, T_n_: current, T_n+1_: next, T_n−1_, T_n−2_: 2 previous). SINT—sinus tachycardia, AVRT—atrioventricular reentrant tachycardia, AFL—atrial flutter.

**Figure 7:**
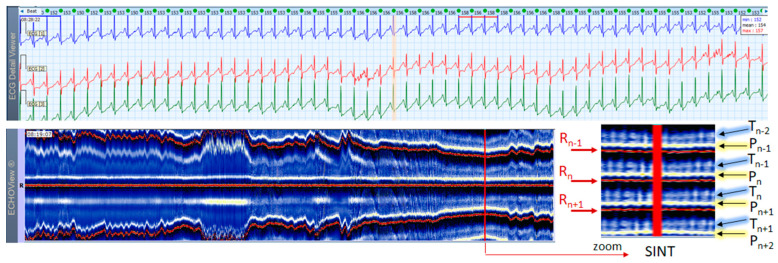
A 3-lead Holter ECG strip (22 s, 25 mm/s) and ECHOView image (ECG lead 1, height = 1500 ms, width resolution = 80 beats/25 mm) of a 21-year old female patient with SINT. The two pages (ECG—top and ECHOView—bottom) are aligned with vertical lines in the highest rate SINT (max HR = 158 bpm read from instant HR values above each QRS complex in the ECG page). In ECG, the atypical morphology of the P-wave (narrow-based, tall, and sharp) and its superimposition on the previous T-wave requires differentiation from an atrial tachycardia. In ECHOView (original and zoomed), the P-wave is a visible blue-white horizontal band (P_n_), preceding the red R-wave line (R_n_), which confirms the sinus rhythm. ECHOView color map: Yellow-white trace (P-wave of the atrial depolarization, P_n_: current, P_n+1_, P_n+2_: 2 next, P_n−1_: previous); Red traces (R-peak of ventricular depolarization, R_n_: current, R_n+1_: next, R_n−1_: previous); Blue-white traces (T-wave of ventricular repolarization, T_n_: current, T_n+1_: next, T_n−1_, T_n−2_: 2 previous). SINT—sinus tachycardia.

**Figure 8:**
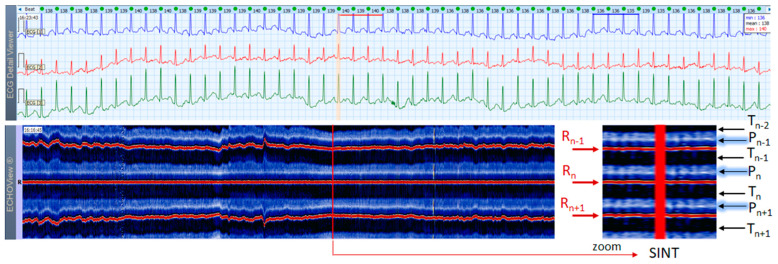
A 3-lead Holter ECG strip (22 s, 25 mm/s) and ECHOView image (ECG lead 1, height = 1500 ms, width resolution = 80 beats/25 mm) of a 38-year old male patient with SINT. The two pages (ECG—top and ECHOView—bottom) are aligned with vertical lines in the highest rate SINT (max HR = 148 bpm read from instant HR values above each QRS complex in the ECG page). In ECG, the atrial depolarization before the R wave is barely visible in the ECG channels and differentiation of SINT from AVNRT in this case is difficult. In ECHOView (original and zoomed), the P-wave is a visible white horizontal band (P_n_), preceding the red R-wave line (R_n_), which confirms the sinus rhythm. ECHOView color map: Blue-white traces (P-wave of atrial depolarization, P_n_: current, P_n+1_: next, P_n−1_: previous); Red traces (R-peak of ventricular depolarization, R_n_: current, R_n+1_: next, R_n−1_: previous); Black traces (T-wave of ventricular repolarization, T_n_: current, T_n+1_: next, T_n−1_, T_n−2_: 2 previous). SINT—sinus tachycardia, AVNRT—atrio-ventricular nodal reentrant tachycardia, AFL—atrial flutter.

This section interprets together 3-lead Holter ECG strips and the corresponding ECHOView images (ECG lead 1) of three patients with SINT (Figure 6, Figure 7 and Figure 8). When the HR is too high, it is sometimes difficult to differentiate true SINT, caused by a physiological increase in the sinoatrial node activity from SVTs due to re-entry circuits, increased focal atrial automaticity, or triggered activity. The examples below represent somewhat complex cases from an ECG diagnostic perspective, where SINT mimics atrioventricular reentrant tachycardia (AVRT) (Figure 6), or focal atrial tachycardia, atrioventricular nodal reentrant tachycardia (AVRNT) (Figure 8), AFL with AV conduction 2:1 (Figure 6 and Figure 8) or atrial tachycardia (Figure 7). The general difficulty is the recognition of the sinus P-wave at high cardiac rates, particularly in Holter ECG strips recorded with less number of channels than the standard 12-lead ECG (usually made at rest, when sinus tachycardia is not so common). As shown in one-lead ECHOView images below, the sinus P-wave band in SINT is well seen as a blue-white band. In the three examples, it maintains a constant distance from the red band of the central R-peak, which stands for a stable PR-interval, regardless of beat-by-beat RR-interval change. The latter is well seen in the ECHOView images by three horizontal red traces for three sequential R-peaks, presenting useful information on the long-term HR variation in SINT, which demonstrates patient-specific dynamics in the three examples.

### 3.4. Paroxysmal Supraventricular Arrhythmia Case

**Figure 9:**
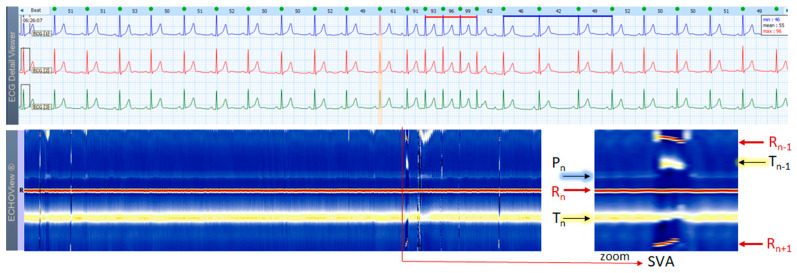
A 3-lead Holter ECG strip (22 s, 25 mm/s) and ECHOView image (ECG lead 1, height = 1500 ms, width resolution = 80 beats/25 mm) of a 44-year old male patient presenting a short SVA episode, probably an accelerated nodal rhythm. The two pages (ECG—top and ECHOView—bottom) are aligned with vertical lines in the zone of interest, including SVA (HR = 91–99 bpm read from instant HR values above each QRS complex in the ECG page). In ECG, the P-wave is hidden inside the QRS complex or in the T-wave (best seen as a ‘notching’ of the T-wave of the last arrhythmia complex). In ECHOView, this arrhythmia is represented as a distinctive vertical line, clearly distinguishable from the adjacent sinus rhythm periods due to notable RR-interval change, leading to dislocation of red (R_n−1_, R_n+1_) and yellow (T_n−1_) bands. The zoomed ECHOView shows important details, including a discontinuity of the P-wave band (P_n_) for the time of the arrhythmia and slight changes in the T-wave band (T_n_), probably due to superimposition with the atrial excitation. ECHOView color map: Blue-white trace (P-wave of atrial depolarization, P_n_: current); Red traces (R-peak of ventricular depolarization, R_n_: current, R_n+1_: next, R_n−1_: previous); Yellow-while traces (T-wave of ventricular repolarization, T_n_: current, T_n−1_: previous). SVA—supraventricular arrhythmia.

Figure 9 shows 3-lead Holter ECG strips and the corresponding ECHOView image (ECG lead 1) of a representative case of paroxysmal SVA (a short 4-beat rush of accelerated nodal rhythm HR = 91–99 bpm) that is not a tachycardia (HR < 100 bpm). Since the excitation originated in the AV junction and propagated simultaneously to the atria and the ventricles, in ECG the P-wave is hidden inside the QRS complex or in the T-wave (best seen as a ‘notching’ of the T-wave of the last arrhythmia complex). In ECHOView, this arrhythmia is represented as a distinctive color vertical line, clearly distinguishable from the adjacent sinus rhythm periods due to the shortening of RR-intervals. Zoomed ECHOView clearly shows some details, including a discontinuity of the P-wave band for the time of the arrhythmia and slight changes in the T-wave band, probably due to superimposition with the atrial excitation. 

### 3.5. Paroxysmal Supraventricular Tachycardia Cases

**Figure 10:**
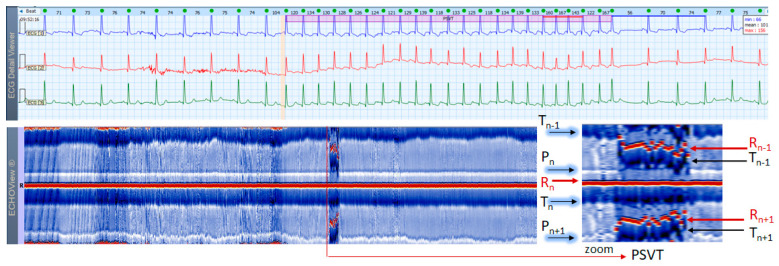
A 3-lead Holter ECG strip (22 s, 25 mm/s) and ECHOView image (ECG lead 1, height = 1500 ms, width resolution = 80 beats/25 mm) of a 62-year old female patient with PSVT (10 s). The two pages (ECG—top, ECHOView—bottom) are aligned with vertical lines in the zone of interest, including PSVT (HR = 118–167 bpm read from instant HR values above each QRS complex in the ECG page). In ECG, HR >100 bpm, changeable morphology of the atrial non-sinus P-wave (>3 different shapes) and varying RR intervals are consistent with a multifocal atrial tachycardia. AVRT or atypical AVNRT cannot be excluded either. In ECHOView, the PSVT run is marked by the appearance of two red R-wave bands of previous and next beats (R_n−1_, R_n+1_) due to RR-intervals shortening, as well as an interruption of the blue-white sinus P-wave band (P_n_). The zoomed ECHOView clearly shows some important details in the dark-blue T-wave band (T_n_), which is also interrupted by different-sized dark spots, representing the multifocal origination of atrial excitations, superimposing on the T-wave. ECHOView color map: Blue-white traces (P-wave of atrial depolarization, P_n_: current, P_n+1_: next); Red traces (R-peak of ventricular depolarization, R_n_: current, R_n+1_: next, R_n−1_: previous); Dark-blue traces (T-wave of ventricular repolarization, T_n_: current, T_n−1_: previous, T_n+1_: next). PSVT—paroxysmal supraventricular tachycardia, AVNRT—atrioventricular nodal reentrant tachycardia, AVRT—atrioventricular reentrant tachycardia.

**Figure 11:**
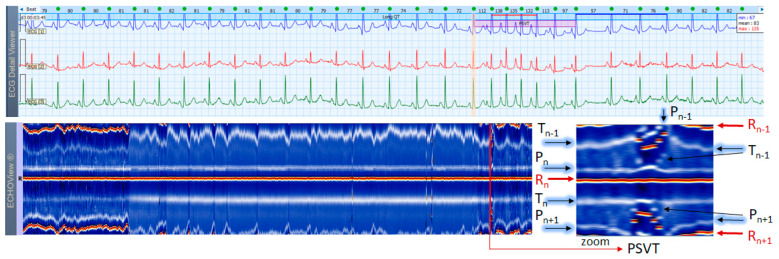
A 3-lead Holter ECG strip (22 s, 25 mm/s) and ECHOView image (ECG lead 1, height = 1500 ms, width resolution = 80 beats/25 mm) of a 46-year old female patient with short PSVT episode (3 s). The two pages (ECG—top and ECHOView—bottom) are aligned with vertical lines in the zone of interest, including PSVT (HR = 112–138 bpm read from instant HR values above each QRS complex in the ECG page). In ECG, PSVT is seen as a 5-beat run of a multifocal tachycardia (≥3 different P-wave morphologies can be seen, the rhythm is irregular). Since all atrial non-sinus P-waves precede R-peak (R_n_) and are very close to the timing of the normal sinus P-wave (P_n_), the zoomed ECHOView shows that the P-wave band is not interrupted, but just displaced anteriorly to the adjacent sinus P-wave line. The arrhythmia episode itself is represented in ECHOView as a distinctive vertical line, well demarcated from sinus rhythm periods. ECHOView color map: Blue-white traces (P-wave of atrial depolarization, P_n_: current, P_n−1_: previous, P_n+1_: next); Red traces (R-peak of ventricular depolarization, R_n_: current, R_n+1_: next, R_n−1_: previous); Blue-white traces (T-wave of ventricular repolarization, T_n_: current, T_n−1_: previous). PSVT—paroxysmal supraventricular tachycardia.

**Figure 12:**
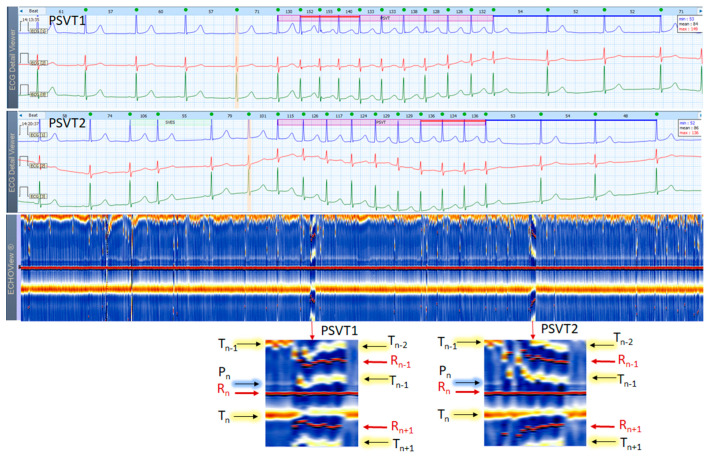
Two 3-lead Holter ECG strips (22 s, 25 mm/s) and corresponding ECHOView image (ECG lead 1, height = 1500 ms, width resolution = 80 beats/25 mm) of a 37-year old female patient with frequent SVES and two short PSVT episodes (4.2 s): PSVT1 (HR = 126–155 bpm) and PSVT2 (HR = 115–136 bpm, read from instant HR values above each QRS complex in the ECG page). In ECG, at first glance the SVT looks similar to an AVNRT (a paroxysmal, narrow-QRS complex tachycardia, starting after an SVES, HR looks regular, and no atrial P-waves are visible). A closer look, however, may identify a subtle ‘notching’ on the top of the T-wave in lead 2 of PSVT1 strip. If we accept it as a retrograde P-wave with an RP interval >90 ms, then an AVRT must be excluded (a concealed accessory pathway with retrograde conduction). A focal AT is also possible. In zoomed ECHOView, the P-wave band during PSVT is interrupted and the P-wave segment (P_n_) is embedded in the T-wave (T_n−1_), which supports the necessity to exclude an AVRT with a concealed retrograde pathway. In a typical ‘slow-fast’ AVNRT, P-waves are hidden in the QRS complex, and in a ‘fast-slow’ AVNRT, P-waves are visible between the QRS and T-wave (ST-segment). In these cases, the P-wave segment is expected to be found within or very close to the R-wave band in EchoView, which is not seen in this patient. ECHOView color map: Blue-white trace (P-wave of atrial depolarization, P_n_: current); Red traces (R-peak of ventricular depolarization, R_n_: current, R_n+1_: next, R_n−1_: previous); Orange-white traces (T-wave of ventricular repolarization, T_n_: current, T_n−1_: previous, T_n+1_: next). PSVT—paroxysmal supraventricular tachycardia, SVES—supraventricular extrasystole, AVNRT—atrioventricular nodal reentrant tachycardia; AVRT—atrio-ventricular reentrant tachycardia, AT—atrial tachycardia.

This section interprets 3-lead Holter ECG strips and the corresponding ECHOView images (ECG lead 1) of three patients presenting short runs (≤10 s) of paroxysmal supraventricular tachycardia (PSVT) at the background of predominant NSR for most of the record (Figure 10, Figure 11 and Figure 12). These arrhythmias could occur at any age and are more common among women. The ‘slow-fast’ AVNRT is the most common form of PSVT, particularly in otherwise healthy hearts. It is caused by a reentrant circuit, created between two pathways (‘slowly’ and ‘fast’ conducting pathways) in or around the AV node. When the HR is too high, it is sometimes difficult to differentiate PSVT from high-rate SINT, especially in people without underlying cardiac or non-cardiac diseases. In patients with overt cardiac diseases or other pathological conditions (for example hyperthyroidism), PSVT must be differentiated not only from sinus tachycardia (common in such conditions) but also from high-rate AFL/AF. ECHOView color maps in such cases present quite distinctive patterns of PSVTs compared to NRS and SINT (Figure 6, Figure 7 and Figure 8). The beginning and the end of the arrhythmia can be identified at a glance (Figure 10, Figure 11 and Figure 12). In addition, ECHOView may facilitate the differentiation of the different PSVT subtypes. In summary, ECHOView improves the reading of Holter recordings with PSVT by saving time to visually locate the arrhythmia episodes and refine the diagnosis of these typically short events. 

### 3.6. Atrial Fibrillation and Flutter Cases

**Figure 13:**
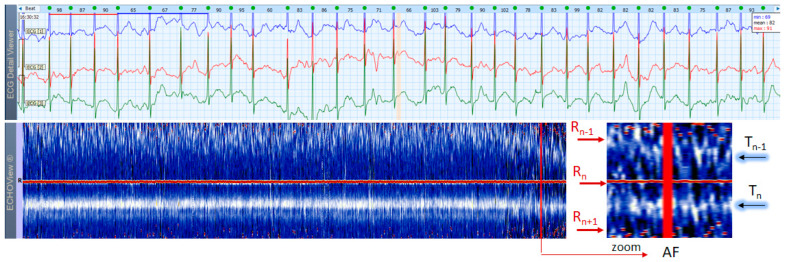
A 3-lead Holter ECG strip (22 s, 25 mm/s) and ECHOView image (ECG lead 1, height = 1500 ms, width resolution = 80 beats/25 mm) of a 68-year old male patient with persistent AF. The two pages (ECG—top and ECHOView—bottom) are aligned with vertical lines. In ECG, the typical AF rhythm irregularity is visible (min–max HR = 60–103 bpm read from instant HR values above each QRS complex in the ECG page). However, the atrial f-waves are not readily visible due to a notable isoelectric line drift possibly in conditions of patient movements. In ECHOView, no distinctive blue-white band typical for the sinus-rhythm P-waves or atrial excitations in SVTs can be seen above the red R-wave line (R_n_), which confirms AF. Instead, the blue-white band of ventricular repolarization (T_n_) following the R-wave can be seen below the red line (R_n_). Due to AF rhythm irregularity, the T-waves of the previous beats (T_n−1_) appear at irregular intervals before R-waves (R_n_), which are seen as scattered blue-white lines above the red line (R_n_). ECHOView color map: Red traces (R-peak of ventricular depolarization, R_n_: current, R_n+1_: next, R_n−1_: previous); Blue-white traces (T-wave of ventricular repolarization, T_n_: current, T_n+1_: next, T_n−1_: previous). AF—atrial fibrillation.

**Figure 14:**
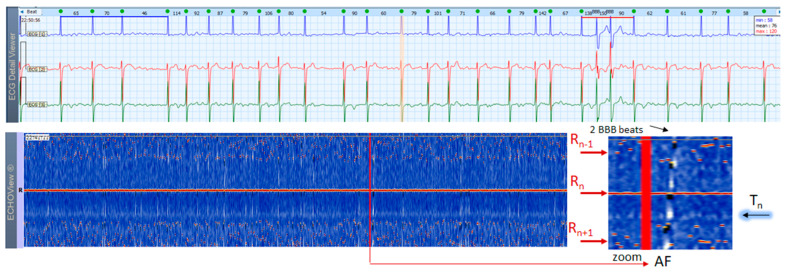
A 3-lead Holter ECG strip (22 s, 25 mm/s) and ECHOView image (ECG lead 1, height = 1500 ms, width resolution = 80 beats/25 mm) of a 84-year old male patient with recurrent persistent AF. The two pages (ECG—top and ECHOView—bottom) are aligned with vertical lines. In ECG, the typical AF rhythm irregularity is visible (min–max HR = 46–150 bpm read from instant HR values above each QRS complex in the ECG page). The fibrillation ‘f’ waves are larger than usual—this type of AF is often regarded as ‘gross AF’. In such patients, differentiation of AF from AFL with variable atrioventricular conduction or from multifocal AT might not be so easy, which could affect the therapeutic decision about restoration and maintenance of sinus rhythm. In ECHOView, no distinctive blue-white band typical for the sinus-rhythm P-waves or atrial excitations in SVTs can be seen above the red R-wave line (R_n_). Instead, randomly distributed white dots (above and below R_n_ line) are noted to probably depict the ‘f’ waves. Due to AF rhythm irregularity, the R-peaks of previous and next beats (R_n−1_, R_n+1_) appear at irregular intervals, which are well seen as scattered red dots above and below the red line of the central R-wave (R_n_). This is the typical ECHOView pattern, which confirms AF. The two wide QRS complexes are probably supraventricular excitations (part of AF) with a functional BBB due to the so-called ‘Ashmann’ phenomenon (aberrant ventricular conduction due to a change in QRS cycle length). ECHOView color map: Red trace and dots (R-peak of ventricular depolarization, R_n_: current, R_n+1_: next, R_n−1_: previous); Blue-white trace (T-wave of ventricular repolarization, T_n_: current); random blue-white dots (atrial fibrillatory f-waves). AF—atrial fibrillation, AFL—atrial flutter, SVT—supraventricular tachycardia, AT—atrial tachycardia, BBB—Bundle Branch Block.

**Figure 15:**
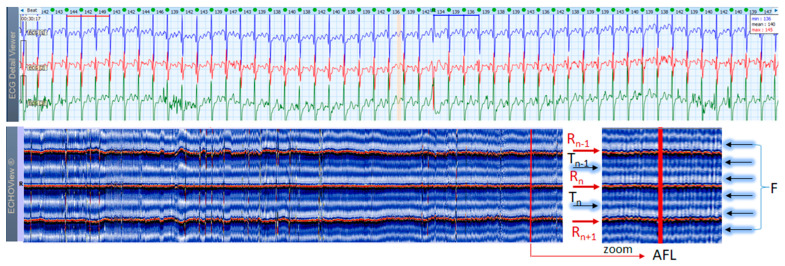
A 3-lead Holter ECG strip (22 s, 25 mm/s) and ECHOView image (ECG lead 1, height = 1500 ms, width resolution = 80 beats/25 mm) of a 61-year old male patient with AFL with atrial-ventricular conduction 2:1. The two pages (ECG—top and ECHOView—bottom) are aligned with vertical lines in high-rate AFL (min–max HR = 138–146 bpm read from instant HR values above each QRS complex in the ECG page). In ECG, the deflections of the isoelectric line (‘F’ waves) are not as typically described (‘saw-like’)—a focal/multifocal AT may have similar appearance. The ECHOView map shows rapid and regular HR typical for AFL, seen as stable red bands of previous (R_n−1_) and next beats (R_n+1_). Cardiac repolarization is recognized as a blue-white band, which follows sequential R-peak at a queasy-equal delay (T_n_: is the band of the current beat, T_n−1_ is the band of the previous beat). The F-waves of the macro-reentrant excitations of the atria can be seen as two blue-white bands equally spaced between any two adjacent R-peaks. The long-term ECHOView map shows certain changes in the F-wave conduction, where the first F-wave and T-wave are overlapping (most left part) but further diverge and form separate blue-white traces (most right part). This pattern is quite different than the single P-wave band in SINT and AT, or randomly distributed f-wave white dots in AF. ECHOView color map: Blue-white traces (F-waves of atrial depolarization, T-wave of ventricular repolarization, T_n_: current, T_n−1_: previous), Red traces (R-peak of ventricular depolarization, R_n_: current, R_n+1_: next, R_n−1_: previous). AFL—atrial flutter, AF—atrial fibrillation, SINT—sinus tachycardia, AT—atrial tachycardia.

AF is the most common arrhythmia among humans (1 of 4 will have at least 1 episode of this arrhythmia in his/her life). This rhythm disorder and the less common AFL are usually not difficult to be recognized in standard 12-lead ECG strips if the ventricular rate is not too high and the fibrillation ‘f’ waves or flutter ‘F’ waves are visible in at least one lead. However, if HR is very high and irregularity of the rhythm, and fibrillations (in AF) or ‘saw-like’ fluctuations of the isoelectric line (in AFL) are not visible, it could be difficult to say what kind of arrhythmia the patient has. In addition, in many patients these arrhythmias are transitory and sought by Holter ECG/event recorders, which records frequently have a lot of artifacts due to physical activities and other factors. In such cases, ECHOView may help to recognize AF/AFL arrhythmias because they have a very characteristic pattern on the color map, as shown in Figure 13, Figure 14 and Figure 15. The notable behavior of AF is the absence of a P-wave band and scattered dots of R-wave peaks (Figure 13 and Figure 14), while AFL has presence of several F-wave bands and stable band of R-wave peaks (Figure 15). Beneficially, these ECHOView maps sufficiently differ from the patterns of other high-rate arrhythmia, such as SINT (Figure 6, Figure 7 and Figure 8), SVA (Figure 9) and PSVT (Figure 10, Figure 11 and Figure 12).

### 3.7. A Case with Transition AF, AFL, SVT

**Figure 16:**
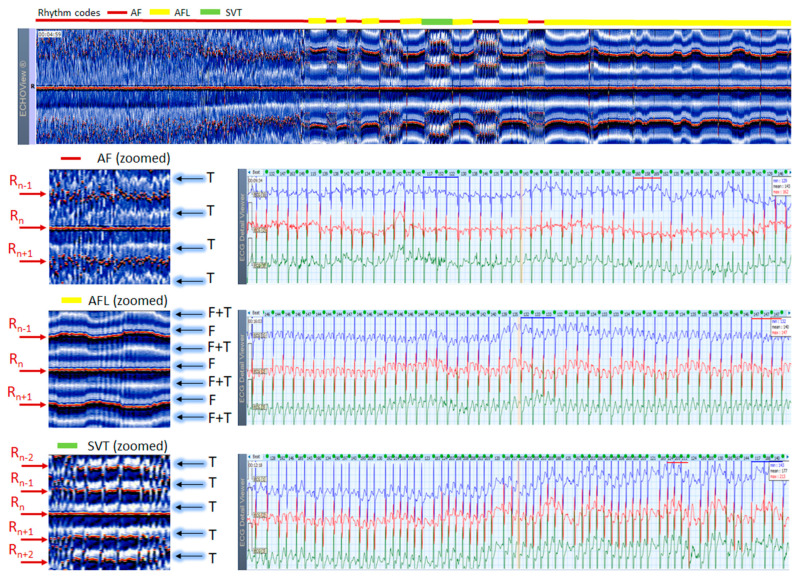
ECHOView image (ECG lead 1, height = 1500 ms, width resolution = 80 beats/25 mm) of a 61-year old male patient with alternating arrhythmia episodes, the period of which is marked at the top of the image by a horizontal bar with rhythm color codes: red (AF), yellow (AFL), green (SVT). Zoomed ECHOView images of selected arrhythmia episodes and their corresponding 3-lead Holter ECG strips (22 s, 25 mm/s) are presented: top—AF, min–max HR = 117–163 bpm; middle—AFL with atrial-ventricular conduction 2:1, min–max HR = 132–147 bpm; bottom—SVT with a characteristic of high-rate multifocal AT, min–max HR = 117–224 bpm. The three arrhythmias have the typical pattern observed in ECHOView for AF (absence of P-wave band and scattered red dots of R-wave peaks), AFL (presence of two blue-white F-wave bands and stable red bands of R-wave peaks), SVT (presence of one blue-white band of ventricular repolarization (suggested superposition with multifocal atrial excitation) and stable red bands of R-wave peaks). In this case, ECHOView is very important for differentiation between the second and third arrhythmia, because at first glance ECG strips seem similar. ECHOView color map: Blue-white traces (F-waves of atrial flutter, F + T: F-waves superimposed with ventricular repolarization); Red traces (R-peak of ventricular depolarization, R_n_: current, R_n+1_, R_n+2_: 2 next, R_n−1_, R_n−2_: 2 previous); Blue-white traces (T-wave of ventricular repolarization). AF—atrial fibrillation, AFL—atrial flutter, AT—atrial tachycardia, SVT—supraventricular tachycardia.

The example in Figure 16 shows Holter ECG strips and ECHOView maps of a patient with alternating arrhythmias: AF, AFL and SVT. In ECG, the differences between arrhythmias are searched in the low-amplitude P-wave, which is difficultly followed in such high-rate arrhythmia (presenting HR from 117 to 244 bpm in that particular case) and isoelectric line drift due to movements. The ECHOView color map demarks the periods of these arrhythmias by their specific patterns with prominent recognition of their start and end instants at a glance. 

### 3.8. Ventricular Arrhythmia Case

**Figure 17:**
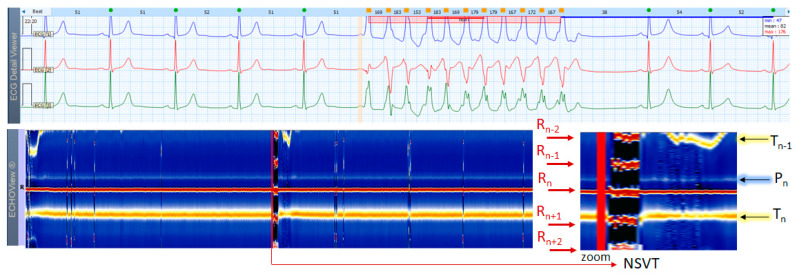
A 3-lead Holter ECG strip (22 s, 25 mm/s) and ECHOView image (ECG lead 1, height = 1500 ms, width resolution = 80 beats/25 mm) of 44-year old male patient with NSR and a short NSVT episode (4 s). The two pages (ECG—top and ECHOView—bottom) are aligned with vertical lines in the zone of interest including NSVT (min–max HR = 153–183 bpm read from instant HR values above each QRS complex in the ECG page). Both, ECG and ECHOView present distinctive NSVT pattern morphology compared to NSR. During NSVT, the ECHOView image shows: (i) interruption of the blue-white P-wave band (P_n_) of NSR due to the lack of normal atrial excitation; (ii) interruption of the orange-white T-wave band (T_n_) of NSR due to ST-segment depression and an inversion of the T-wave electric axis; (iii) appearance of four red R-wave bands of previous and next beats (R_n−2,_ R_n−1_, R_n+1_, R_n+2_) due to RR-intervals shortening; (iv) widening of the central R-wave band due to QRS complex enlargement. The NSVT image appears as horizontal red and black bands, corresponding to strongly positive R-peaks and strongly negative T-waves. During NSR, the occasional vertical line disruptions of the ECHOView map correspond to single VES similar to ones in the NSVT rhythm. ECHOView color map: Blue-white traces (P-wave of atrial depolarization, P_n_: current); Red traces (R-peak of ventricular depolarization, R_n_: current, R_n+2,_ R_n+1_: 2 next, R_n−1_, R_n−2_: 2 previous); Orange-white traces (T-wave of ventricular repolarization, T_n_: current, T_n−1_: previous). NSVT—non-sustained ventricular tachycardia, NSR—normal sinus rhythm, VES—ventricular extrasystole.

The example in Figure 17 shows a Holter ECG strip and the corresponding ECHOView map, including a short rush of NSVT in a patient with NSR. NSVT is a broad-QRS complex tachycardia that is usually easily recognized in ECG strips. However, SVT in some patients with functional or fixed branch block could also be presented in ECG as a broad-QRS complex tachycardia, making differentiation from a VT difficult. In ECHOView, VT has a quite distinguishable pattern with red/black bands, corresponding to the strongly positive/negative R/T waves, respectively. The ventricular origin of this high-rate arrhythmia can be confirmed by different ECHOView observations, such as the absence of P-wave band due to the lack of normal atrial excitation; inversion of the color of the T-wave band due to ST-segment depression and inversion of the T-wave electric axis; appearance of several R-wave bands due to RR-interval shortening; widening of the central R-wave band due to QRS complex enlargement. 

Although diagnosing VT may not be a major problem in ECG, reliably detecting typically brief episodes of NSTV in a 24-h Holter ECG is a challenge that compressed ECHOView images can greatly assist.

## 4. Discussion

In this study, we present some pilot data from clinical research focused on the assessment of the compression and diagnostic potential of a relatively new and still not well-investigated ECG modality—ECHOView color maps, provided by commercial software for Holter ECG analysis. We tested this technique for the identification and differentiation of different rhythm disorders—SINT, SVES, VES, SVTs, AF, AFL, and VT and presented examples of patients, in whom Holter ECG diagnosis has been difficult (for objective and subjective reasons), and ECHOView facilitated rhythm recognition. 

### 4.1. Practical Aspects of ECHOView: What Is Known and What Remains Uncertain?

The compression ratio statistics (median, quartile range) showed that one Holter ECG recording is reviewed with about 67 (59–74) times fewer number of ECHOView pages than standard grid ECG pages. However, the real impact of interpreting ECHOView images on the reading time of a 24 h (or longer) Holter recording requires a separate clinical study, with a design focused on this issue. The study in this article was not aimed to specifically measure the reading time, so our opinion for its substantial shortening is subjective. 

We underline two main advantages of the compressed ECHOView information:

- Reduction of Holter records revision time, based on three additive components: (1) time for preprocessing (noise parts exclusion); (2) time for visual finding of events; (3) time for making diagnostic conclusions. Although the total Holter ECG revision time was not measured in this study, we found that the ECHOView compression ratio significantly reduced the time for identification of events of interest. It made it possible for a 24.5-min period to be analyzed at a glance. The conventional reading of Holter ECG strips, each of them 22 s long, takes much longer. Additionally, after event localization, combined ECHOView and ECG assessment were helpful in reducing the time for diagnostic conclusions.
- Keeps the clinician’s attention focused on the record for the entire monitoring period: a 24 h Holter ECG recording is presented in just 51 ECHOView pages instead of 3491 ECG pages on average. With ECHOView large epochs of the record can be encompassed, compared, and comprehended at a glance.

### 4.2. ECHOView in the Diagnosis of Different Arrhythmia Types

#### 4.2.1. Supraventricular and Ventricular Extrasystoles

In real clinical practice, many patients with complaints of palpitations/irregularity of the heart rate/skipped beats are found to have just SVES or VES, usually not life-threatening and not requiring treatment unless too frequent (VES > 10% of all complexes) or impairing the quality of life. ECHOView can facilitate/improve the quantification of the total number of extra beats and their timing. Using the ECHOView modality we found ectopic beats in a large part of our study population (SVES in 84%, VES in 78%). 

#### 4.2.2. Supraventricular Tachyarrhythmias: Sinus Tachycardia, Supraventricular Tachycardias, Atrial Fibrillation and Flutter

ECHOView interpretation turned out to be very important for some of our patients with episodes of supraventricular rhythm disorders such as SINT, SVT, AF and AFL. These arrhythmias are very common among the general population, affecting both younger and older people, with or without underlying cardiac injury [1,2,3,4,5,6,7]. A lot of non-cardiac conditions such as thyroid dysfunction, chronic lung diseases, electrolyte disturbances, infections (including COVID-19), etc. could also ‘unlock’ SVAs [1,2,8]. The diagnosis is based mostly on non-invasive ECG methods, mainly standard 12-lead ECG and 24–72 h Holter ECG monitoring [1,2,3,4,5,6,7,8,9,10]. Standard 12-lead ECG records are obtained in rest and their quality is usually much better than Holter ECG records, but conventional ECGs are not the ideal tool for diagnosing arrhythmias. They present time frames of about 10–20 s and many patients with SVAs could be missed because arrhythmias are often transitory [1,2,3,4,5,8]. For this reason, diagnosis is usually obtained by Holter ECG monitoring or event recorders [1,2,3]. However, most of these devices record less number of channels (1 to 3) in which P-waves of the normal sinus rhythm may not be demonstrated [1,2,3,4,5,6]. In addition, external factors such as movements, posture, skin contact of the electrodes, etc. may exert significant influence on the quality of the records and the morphology of ECG components, thus making the detection of P-waves even more difficult [1,8,17]. One of the most common ECG challenges is the differentiation of a high-rate sinus tachycardia from other SVTs (AVNRT, AVRT, focal AT) and sometimes from high-rate AF/AFL. Physical activities, emotions, panic attacks, pain/severe discomfort, febrility, etc. may cause expressed sinus tachycardia with complaints indistinguishable from a true tachyarrhythmia [3,5,9]. The ECG leads to shortening of the RR intervals, the impossibility of recognizing regularity/irregularity of the rhythm, and identifying/distinguishing the normal sinus P-wave from pathological P-waves of AT, F waves of AFL and f-waves of AF could make diagnosis uncertain, particularly if the patient has already had arrhythmias or risk factors for the development of them [5,7,9]. In this article we present several cases in which patient’s complaints and the Holter ECG records required differentiation between SINT, SVT and AF/AFL. In our cases, ECHOView facilitated the accurate diagnosis clearly disclosing the P-wave in SINT (Figure 6, Figure 7 and Figure 8) and the pathological atrial excitations in cases of true tachyarrhythmias SVT (Figure 10, Figure 11, Figure 12 and Figure 16), AF (Figure 13, Figure 14 and Figure 16) and AFL (Figure 15 and Figure 16) but also the contrastive ventricular pattern of NSVT. As the prognostic significance and therapeutic approach to arrhythmias can vary greatly [40], such as the need for oral anticoagulation in AF/AFL [1,2], the advanced perspective of ECHOView is an important tool to improve risk assessment and therapeutic decision-making.

### 4.3. ECHOView: Summary of the Principles of Imaging

ECHOView modality discloses clearly in a color-coded pattern the presence and morphology of different ECG waves in sequential beat intervals. Thanks to the highly distinctive color map (black-blue-white-orange-red), the ECG waves can be easily discerned and their alignment to the central R-peak can be tracked in long duration ECHOView pages (mean duration of 24.5 min per page). This is especially important for the traditionally difficult recognition of the P-wave, which in all example cases is visible as a blue-white horizontal trace above R-peaks when present in NSR (Figure 4, Figure 5, Figure 9, Figure 10 and Figure 11), SINT (Figure 6, Figure 7 and Figure 8) and PSVT (Figure 10, Figure 11 and Figure 12). Similarly, a series of vertically displaced white traces are recognized for the F-waves in AFL (Figure 15 and Figure 16). On the contrary, a white horizontal P-wave trace is missing in NSVT (Figure 17) and AF (Figure 13, Figure 14 and Figure 16), where f-waves are discerned as randomly located light spots. The presence or absence of the P-wave in SVES and VES beats is difficult to be recognized in compressed ECHOView pages unless the area of the ectopic beat is zoomed as in Figure 4 and Figure 5 (right). Nevertheless, the identification of SVES/VES is possible by observations of the red dots (R-peak) and yellow/orange dots (T-peak) visible as occasional events in the blue horizontal area between T_n−1_ and P_n_ (Figure 4 and Figure 5). Additionally, VES are associated with a change in the QRST waveform compared to NSR beats, which is recognized on the ECHOView color map as a contrasting color change, e.g., in Figure 5 from blue to black (during the negative T-wave of VES). Whereas the inspection of such color changes for single VES beats requires a zoomed ECHOView image (Figure 5), the recognition of NSVT episodes including multiple VES beats seems easier in the compressed ECHOView page (Figure 17). However, our observations of NSVT are limited because these are rare events in our Holter ECG population, observed in only 3 patients with short rushes ≤ 11 beats.

The color-coded ECG amplitudes of sequential beats produce an intuitive trace of different intervals (P_n_R_n_, R_n_T_n_, R_n−1_R_n_, R_n_R_n+1_, etc.) in a visible image (width × height = 1740 beats × 1500 ms) within one ECHOView page. Without a specific measurement, this visual effect resembles the rendering of one-dimensional time trends of automatically measured ECG intervals. These trends show stable P_n_R_n_ and R_n_T_n_ intervals during the NSR part of the examples in Figure 4, Figure 5, Figure 6, Figure 7, Figure 8, Figure 9, Figure 10, Figure 11 and Figure 12. The trends are also informative for the RR-intervals (R_n−1_R_n_, R_n_R_n+1_) on a beat-by-beat basis that is helpful for identifying long-term HR changes in high-rate SINT (Figure 6, Figure 7 and Figure 8) and AFL (Figure 15), as well as rhythm, transitions NSR→ paroxysmal SVA (Figure 9), NSR→PSVT (Figure 10, Figure 11 and Figure 12), NSR→NSVT (Figure 10, Figure 11 and Figure 12), and AF→AFL→ST (Figure 16). 

In summary, ECHOView color maps are a comprehensive visualization tool of a patient’s rhythm that helped the reading of 24 h Holter ECG recordings performed by a cardiologist. For many of our patients, it reduced the time to overview a Holter record and helped to establish the correct diagnosis, particularly when high cardiac rates have been registered. This ECG modality was most useful to differentiate episodes of high-rate SINT from SVTs, where the role of ECHOView for diagnosis confirmation/exclusion was crucial. 

## 5. Limitations

Although important benefits of ECHOView color maps are disclosed for fast reading of Holter ECG recordings, this functionality is accessible only under a software license (copyright by Schiller AG, Switzerland). 

In our study, ECHOView images gave a general impression of the frequency of SVES and VES, but they were not superior to the ECG modality for qualitative evaluation of these common rhythm disorders in the general population. Our results did not disclose any specific ECHOView markers that could improve recognition of the electrophysiological mechanisms of origination of the detected SVTs: reentrant circuit, triggered activity, or increased automaticity which may be of help for the treatment approach. 

The number of patients in our study does not allow extrapolation of our results over the entire population of patients with arrhythmias. Currently, there are no elaborated standards for many of the ECHOView components based on large electrophysiological and clinical studies. Further investigations are needed in this direction.

## 6. Conclusions

This observational Holter ECG clinical study demonstrates how the compressed beat pattern information of the ECHOView diagnostic images can be interpreted for the diagnosis of the patient rhythm taken by a cardiologist. Particular benefit was found in reducing the time for visual finding of short arrhythmia events and the time for taking diagnostic conclusions in 24-h Holter ECG. Example cases from clinical Holter ECG study are presented to demonstrate the facilitated finding and interpretation of the most frequent SVAs (SVES, VES, paroxysmal SVA, SINT, PSVT, AFIB, AFL), but also less frequent VAs (VES and NSVT) observed in the patient population. The ECHOView modality has the potential to improve the cost-effectiveness of the Holter ECG reading process, which is associated with improvement of the diagnostic service and patient care, especially for those with complex arrhythmias. The intuitive perception of the colorful ECHOView beat patterns and their dynamics in different pathologies would also be very attractive for educational purposes. Although these conclusions about the usefulness of ECHOView are made within the framework of visual interpretation of images generated by specific software, these benefits from a cardiologist’s perspective can be transferred to a machine learning perspective. The potential improvement of arrhythmia detection based on ECHOView interpretation by an automated algorithm is a subject of future work.

## Data Availability

Restrictions apply to the availability of these data. Proprietary data were used, which may be available on reasonable request from the corresponding author.
